# Progress in Bacteriology

**Published:** 1904-03-05

**Authors:** 


### Progress in Bacteriology.

**Leishman's Bodies.**—Ross [1] figures a series of these bodies stained by Romanowsky's method. They are of two kinds, free and embedded. The free bodies are elliptic or circular, with a strong outline and with two chromatin masses in the minor diameter of the cells. Sometimes one of these masses seems to bulge beyond the cell wall. Embedded bodies are only found in preparations made intra vitam. They differ from the free bodies in that they are less numerous, and their contour is less plain. The matrix in which they lie is granular, regularly oval in shape, but has no definite outline. Each mass of matrix measuring from 3–8$\mu$ may contain from 1–8 bodies. Ross has never been able to find trypanosomes or fragments of trypanosomes in films containing these bodies. He has not observed them inside the corpuscles. He regards these bodies as the spores of a mature organism represented by the matrix belonging to a new genus of sporozoa. In this he differs from Donovan who regards them as belonging to the piroplasma. Donovan [2] whose early work on this parasite is well known, says that the characteristic symptoms of the disease are enlargement of the spleen and liver, irregular pyrexia, paroxysmal œdema of the feet, congestion of the lungs, occasional subcutaneous hæmorrhages, and cancrum oris. He has obtained specimens by puncture of the spleen and liver during life in sixteen cases. He has also found piroplasma in the blood of two calves. In a later communication Ross,[3] who prefers to call the organism the Leishmania donovani, states that Bentley has found the organism in the spleen in kala-azar during life. He also says that Donovan writes that he is unable to find the parasite in the peripheral blood, where one would expect to see them if they were intracorpuscular. Manson and Low [4] have found the Leishman-Donovan bodies in the blood of two cases of non-malarial febrile splenomegaly. They affirm that the bodies are not intracorpuscular, and do not belong to the piroplasma. Employing Leishman-Romanowsky's stain, and carbol-thionin they have made an exhaustive study of the morphology of the parasite, and their paper is accompanied by an excellent sheet of illustrations. In hanging-drop preparations of the blood they state that the matrix with its encysted bodies appears as a mulberry mass, and that from this mass the spores detach themselves, some to undergo endogenous division, others to pass out of the body. How they pass out of the body is so far a mystery. They have never been found in the peripheral blood or the urine, and although from their presence in the liver it was thought that they might travel by the bile ducts to the intestine, they could not be demonstrated in the fæces. A blood-count showed a diminution of hæmoglobin and a lymphocytosis. Leishman,[5] summarising the results up to date, states that he has found the parasite in the blood of two soldiers recently returned from India and who died of splenomegaly, and that Marchand and Leedingham [6] report their occurrence in a German soldier returned from Pekin. He draws attention to the bodies recently described by Wright [7] in cases of Delhi sore. These, he says, closely resemble the bodies he discovered, and he believes that if the life history of Wright's organisms (which are possibly altered cercomonads) were studied more closely further light might be thrown on the intrasplenic bodies. Leishman adheres to his former belief that the bodies are an involuted stage of a flagellate organism, possibly a trypanosome, and he is able in this matter to add the support of Marchand and Leedingham. Probably Laveran's conception of their being piroplasma intracorpuscular bodies will have to be abandoned.

**Rheumatoid Arthritis.**—A bacteriological examination of a case of acute rheumatoid arthritis is recorded by Gask.[8] Although the disease followed childbirth there was no suspicion of sepsis, no heart lesion, no leucocytosis, and the administration of salicylates did not control the disease. The knee-joint was aspirated, and 5 ccs. of slightly turbid fluid withdrawn. Films from this fluid showed cocci and streptococci. A growth was obtained on blood agar and was of a streptococcal type. Some of the fluid was incubated for four days in a sterile pipette by itself, and this, when inoculated into broth, gave rise to a growth of streptococcus, with many diplococcal forms. There was slight formation of acid. Milk was not coagulated. No joint lesions or rheumatic manifestations were produced in a mouse and a rabbit inoculated from the broth cultures; and the rabbit, which was killed later, showed no lesion. The organism was apparently a streptococcus pyogenes. Reviewing recent work on the bacteriology of this disease, we may recall that Schüller found in the synovial fluid of joints affected with rheumatoid arthritis a bacillus measuring 2·6 μ by 0·75 μ. This organism grew on agar as a greyish white film, and liquefied gelatin forming a white mass. It stained readily with the anilin dyes, but was easily discolorised. It showed polar bodies, and when introduced into the joints of animals it excited non-suppurative lesions resembling the changes found in rheumatoid arthritis. Bannantyne, Wohlmann, and Blaxall described a not unsimilar organism, a diplococcus or polar-staining bacillus measuring 2 μ by 0·75 μ, staining with fuchsin or methylene blue, but not with gram. In beef-broth tubes it formed floating minute colonies in four days, and sub-cultures on agar formed a translucent film. It grew well in milk. This organism undoubtedly corresponds to that subsequently described by Chauffard and Ramond, who isolated it from the joint fluids, or lymph glands in the neighbourhood of joints affected with rheumatoid arthritis. Inoculation experiments were not successful, apparently because most animals are insusceptible to the disease.

[1] Brit. Med. Jour., Nov. 28, 1903. [2] Ibid. [3] Brit. Med. Jour., Jan. 16, 1904. [4] Ibid., Jan. 23, 1904. [5] Ibid., Feb. 6, 1904. [6] Lancet, Jan. 16, 1904. [7] Jour. Med. Research, Vol. X., No. 3, p. 472. [8] Trans. Path. Soc., 54, 1903.

